# *In vitro* Macro-qualitative Comparison of Three Enamel Stripping Procedures: What is the Best Shape We can get?

**DOI:** 10.5005/jp-journals-10005-1465

**Published:** 2017-02-27

**Authors:** Nahla Nassif, Mona N Gholmieh, Elia Sfeir, Ayman Mourad

**Affiliations:** 1 Assistant Professor, Department of Pediatric Dentistry, School of Dentistry Lebanese University, Beirut, Lebanon; 2 Associate Professor, Department of Pediatric Dentistry, School of Dentistry Lebanese University, Beirut, Lebanon; 3 Professor, Department of Pediatric Dentistry, School of Dentistry Lebanese University, Beirut, Lebanon; 4 Associate Professor, Department of Mathematics, Faculty of Sciences, Lebanese University, Beirut, Lebanon

**Keywords:** Interdental stripping, Laboratory research, Proximal shape, Reproximation.

## Abstract

**Aim:**

Interdental stripping is a common clinical procedure in orthodontic therapy, by reshaping the proximal contacts. Handheld abrasive strips have been criticized as time-consuming process. Metallic strip system, diamond disk, or segment disks have become increasingly popular. The aim of this study is to evaluate the morphological aspects of remodeled dental surfaces so as to conclude which of the different techniques (disk, bur, or strip) used to reduce the mesiodistal diameter is the best to reproduce the initial contour of the proximal surface of the tooth.

**Materials and methods:**

Seventy-nine pieces (“teeth”) were prepared from permanent healthy teeth (premolars and molars) extracted for orthodontic reasons. They were mounted on a stand resembling the position of the natural teeth in a mild crowded dentition. The “teeth” are divided into three groups as follows: group S (strip): 26 “teeth,” group D (disk): 25 “teeth,” group B (bur): 28 “teeth.” In order to study the changes, these prepared “teeth” are macro-photographed in groups of 5 before and after proximal grinding.

**Results:**

The “teeth” contours have been identified using piecewise cubic Hermit polynomials. The change in the contour has been traduced in terms of the change of curvature in the “teeth” contours. We used the z-test in order to find the confidence interval for the proportion of the class “+” for each of the techniques B, S, and D. With confidence level of 95%, we obtained the following confidence intervals:

B = (0.6943; 0.9057); S = (0.9093; 1.0138); D = (0.6184; 0.8616)

These results can be interpreted, as the technique S is significantly much better than the other two techniques if we aim at conserving the shape of the teeth before and after treatment.

**Conclusion:**

We conclude that the use of a strip for remodeling the proximal surface of a tooth is an optimal technique to preserve the proximal shape of the tooth although it requires more time.

**Clinical significance:**

The use of abrasive strip preserves the best shape of the proximal side. Abrasive strip could be the last step of any proximal reshaping technique.

**How to cite this article:** Nassif N, Gholmieh MN, Sfeir E, Mourad A. *In vitro* Macro-qualitative Comparison of Three Enamel Stripping Procedures: What is the Best Shape We can get? Int J Clin Pediatr Dent 2017;10(4):358-362

## INTRODUCTION

Regardless of the term used, interdental stripping is a common clinical procedure in orthodontic therapy. It aims to improve tooth alignment and long-term maintenance, by reshaping the proximal contacts. Enamel stripping can also be used in the mixed dentition of patients with mild or moderate crowding.^1,2^

In 1944, Ballard^3^ advocated stripping the proximal surfaces of the mandibular anterior segment to correct a lack of harmony in tooth size. A few years later, the stripping technique using metallic strips, followed by polishing and fluoride application for proximal caries preventive measures is described in detail.^4^ Peck and Peck^5^ observed that well-aligned mandibular incisors have significantly lower mesiodistal/faciolingual indices than those of crowded incisors, and recommended stripping for addressing tooth shape deviation. The correction of discrepancies in anterior interocclusal dental arch length might be accomplished by mesiodistal crown reduction or reproxi-mation of the lower anterior teeth in combination with circumferential supracrestal fiberotomy to enhance orthodontic treatment results.^6,7^ Despite the promising results of the preliminary reports, the use of full-arch banding procedures suspended the worldwide development of the stripping concept for decades. It was in the mid-1980s that the air rotor stripping technique of Sheridan attracted worldwide interest from clinicians, and the grinding of the interdental enamel was presented as an alternative to extraction or expansion procedures in cases of mild to moderate crowding.^8,9^ Finally, enamel reshaping was recommended to improve anterior esthetics, i.e., to prevent or reduce interdental gingival retraction (black triangles) that becomes evident after alignment of crowded anterior segments.^10^ Consequently, the clinical use of anterior stripping between 1986 and 2008 has doubled in United States survey of orthodontists.^11^

The mesiodistal enamel reduction is performed by either manual or mechanical method. The early use of handheld abrasive strips has been criticized as a time-consuming process, hardly applicable in the posterior teeth, and leading to irreversible residual furrows on the treated surfaces. Currently, hand-operated strips are reserved for minor enamel removal cases or as introductory or finishing stripping procedures. Alternatively, metallic strip system, diamond disks, or, the most recently developed, segment disks adapted to a shuttle head with oscillation movement have become increasingly popular.^11^

The objective of this study is to evaluate the morphological aspects of remodeled proximal dental surfaces. It aims to determine which of the different techniques used in reducing the mesiodistal diameter is better to reproduce the initial contour of the proximal shape of the tooth.

## MATERIALS AND METHODS

### Survey

In order to determine which techniques to compare, we surveyed by email 135 orthodontists to learn which techniques they use or recommend to reduce the mesiodistal diameter of the teeth during or after an orthodontic treatment. Five categories were defined:

The use of strip, bur, disk, combination of a bur and a strip, or combination of a disk and a strip.

**Fig. 1:**
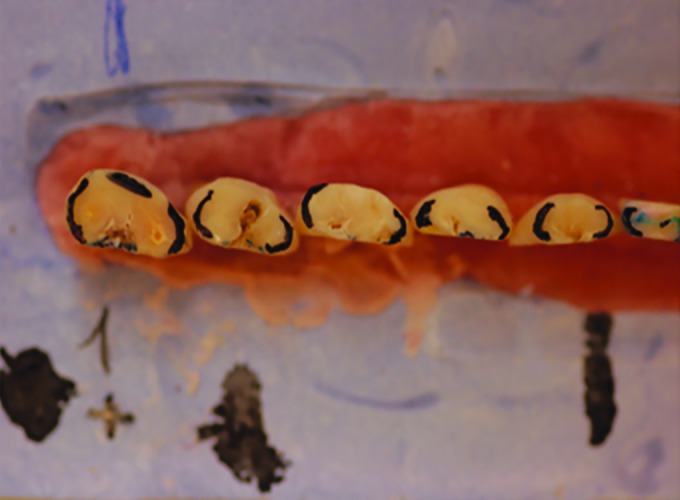
“Teeth” mounted on a hard plaster stand base before grinding procedure

**Fig. 2:**
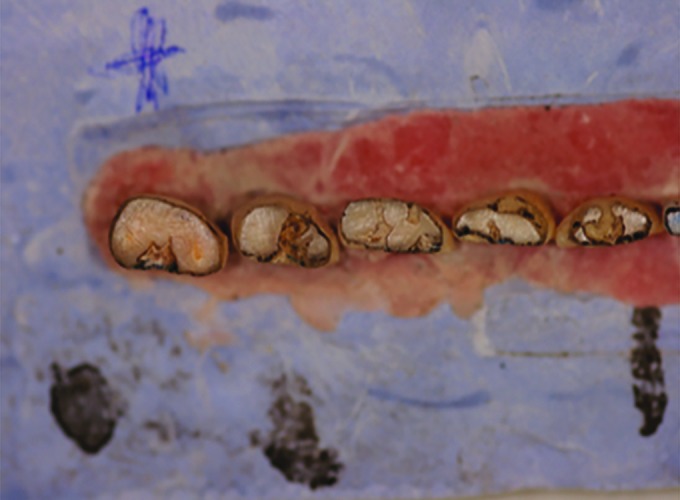
“Teeth” colored by a pencil after grinding procedure

### Experiment

We used 79 permanent healthy teeth (premolars and molars) extracted for orthodontic reasons. Teeth were collected from our department and the oral surgery department of our faculty. Each tooth was cut with a microtome from buccal to lingual direction into two or three parts according to the tooth for simulation of proximal sides of incisor. These divided parts (“teeth”) were chapped with a bur to have a very similar proximal shape and anatomy of the incisors.

These (“teeth”) were fixed with resin, and then mounted on a hard plaster stand base resembling the position of the natural teeth in a mild crowded dentition (Fig. 1).

The incisal edges were all cut at the same level with a separating disk (SS White Separating disks #27), to bring out the thickness of the proximal enamel. Then, the outline of the occlusal surface was drawn with a 0.2 mm thick indelible pencil (Faber-Castel Permanent Multimark 1523 S) to show the thickness of the enamel that should be subsequently grinded.

These prepared “teeth” were macro-photographed (Nikon digital camera D50, Nikon Corp., Japan. Lens EX Sigma 105 mm 1:2, 8 DG macro, Japan) in groups of 5 before and after proximal grinding: The position of each item was identified for consistency in shooting before and after proximal stripping. Before the second macro-photography, occlusal surfaces were colored with a pencil for a better viewing and contrast during the future photography (Fig. 2).

The same operator (N.N.) did all the grinding procedures.

The “teeth” were divided into three groups as follows: Group S (26 “teeth” = 52 proximal sides): The “teeth” were stripped with an abrasive metal strip (6 mm Steel Separating Strips, Becht, Germany). A back and forth movement eliminates the interproximal contact surfaces while removing the already drawn line of enamels’ thickness to strip.^12^

**Figs 3A and B:**
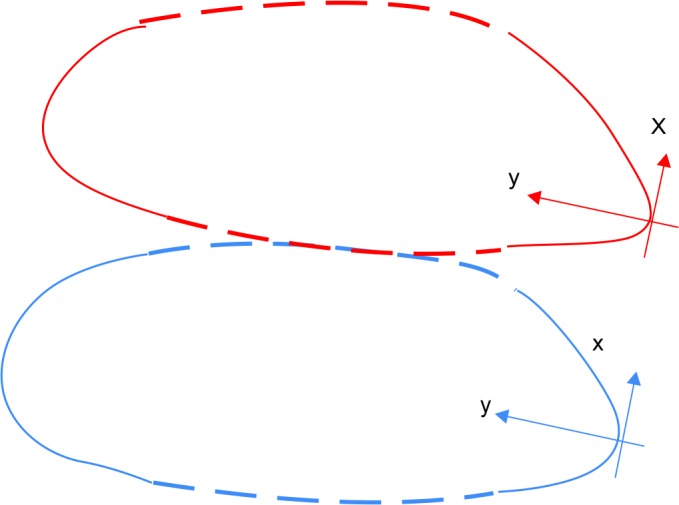
Tooth contour before and after grinding procedure. The local (x, y) axes are shown

Group D (25 “teeth” = 50 proximal sides): The “teeth” were grinded at their contact surfaces as described in group S with a metallic disk (Komet 911H H 204 140, Germany) mounted on blue-ring handpiece.^12^

Group B (28 “teeth” = 55 proximal sides): The “teeth” were grinded with a fine diamond conical bur (Komet 858-016, Germany). The thickness of enamel removed is always respected.^13^

High-resolution photographs were taken before and after grinding. The same camera calibration was used across all pictures acquisitions. In order to study the changes in form before and after the grinding procedure, the “teeth” contours were identified using piecewise cubic Hermit polynomial by a single operator blinded to the grinding method used. The curvature was computed with respect to a local reference of two orthogonal axes (the x-axis and the y-axis) for each contour and its sign noted. The change in the form of “teeth” was defined in terms of the change of curvature in the “teeth” contours. Figure 3 show an example of the choice of the axes before and after grinding.

In this change of reference, the curvature of the contour is given as the second derivative of the variable “y” with respect to the variable “x.” Second-order finite-difference approximation was used to compute accurately the curvature.

Before the “teeth” have been grinded, their corresponding contours look like parabolas and consequently the corresponding curvatures have all positive signs.

After the “teeth” have been grinded, they were classified according to whether the new curvature has conserved the original positive sign or the sign has changed because of the treatment (Table 1).

**Table 1:** **Table 1:** Counts obtained from different techniques. The symbol (+) corresponds to positive curvature, (0) to contours that have straight segments, and (-) for negative curvature

		*+*		*0*		*-*	
B		44		8		3	
S		50		0		2	
D		37		12		1	

## RESULTS

### Survey

Of the 135 orthodontists that were surveyed, all completed the questionnaire: 65 (48%) used a strip, 28 (21%) used a bur, 22 (16%) used a disk, 12 (9%) used a combination of a bur and a strip, and only 8 (6%) used a combination of a disk and a strip. The first three techniques were therefore selected for comparison in the study.

### Statistical Analysis

We used descriptive statistics and observed frequencies of procedure outcomes. Classes “-” and “0” were merged into one class to increase statistical power. We used the z-test to determine the confidence interval for the proportion of the class “+” for each of the techniques S, D, and B.

The proportion of the class “+” for the “teeth” treated by the technique S, lies between 90.93 and 100% with a 95% confidence level. Similarly, the proportion in the case D lies within 61.84 and 86.16%, and in the case B the proportion lies within 69.43 and 90.57%. Moreover, since each of the confidence intervals of the cases B and D is disjoint with the confidence interval of the case S, the techniques B and D are statistically different.

## DISCUSSION

Our results demonstrate that using a strip to reduce the mesiodistal diameter could be superior to using a bur or a disk if aiming at conserving the contour of the teeth. Such technique also appears to be the most adopted by orthodontists. Because many orthodontists have increasingly focused on nonextraction therapy, the documentation and the popularity of enamel reduction has increased. With enamel reduction and cosmetic remodeling, the long-term maintenance of alignment of the mandible incisors and the elimination of black triangles in adults are possible.^2,14^

There are different guidelines regarding the optimal amount of enamel reduction. It varies between 0.4 and 0.6 mm, and a reduction of the enamel by 50% is acceptable.^15-17^

Therefore, controversial studies discussed the consequences of remodeling on teeth. On the one hand, some of them indicate that recontouring by grinding enamel did not increase susceptibility to caries or periodontal disease.^4,6,7,18,19^ On the contrary, some other authors have shown that this procedure can increase the susceptibility of proximal caries.^2,20-23^

Finishing enamel after these procedures is one of the important steps to take into consideration. Many authors studied the surface roughness of the enamel after enamel reduction, and they insist on the polishing step to reach a surface roughness almost similar to that of the corresponding control teeth.^2,23-25^ Consequently, the finer the grain size used for removing enamel, the easier and less time-consuming is the subsequent finishing.

Finally, it is recommended the use of water spray and a low-speed grinding to prevent heat generation during enamel reduction.^9,24^

**Fig. 4:**
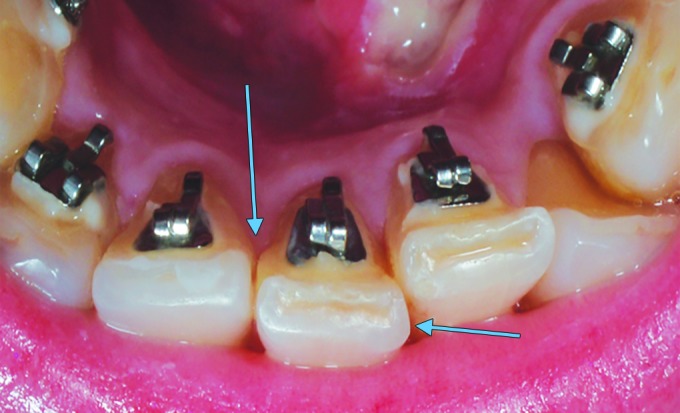
After using the bur, we note a straight interproximal surface

Our statistical analysis has shown that among the different techniques used in this study, the strip one is the best (with confidence level 95%), in the sense that it conserves the contour of the teeth with the highest rate. This result is consistent with our hypothesis although the technique of the strip has been criticized as a time-consuming process compared with the use of a bur or a disk. However, we think that this supposed inconvenient is cons-balanced by the almost exact profile produced and the reduced development of sensitivity, pulp reaction, and exposed dentin.^10^ Moreover, the strip we use is active from one side only so there is no risk to damage the enamel of the neighboring teeth as we might do with the other two techniques especially with the bur. It is also important to mention that the efficiency of a technique is also based on the difficulty of performance of the reduction with a maximum possible precision; more the teeth are crowded, more the reduction is difficult to realize: for that reason in our study, the “teeth” were mounted resembling a mild crowding. The use of a strip permits to control gradually the thickness of the enamel to reduce. Clinically and practically, we can say that with the strip, the proximal walls can be remodeled in an acceptable morphology: the operator can control the “profile” of the proximal surface of the tooth. On the contrary, using the bur and/or the disk alone, without finishing with a strip, may lead to a straight interproximal surface, especially if the operator does not control the technique (Fig. 4). A great care must be taken not to introduce proximal steps when mesiodistal tooth width adjustments are performed.^10^ We believe that the use of the strip prevent the eventual creation of a step.

While our *in vitro* experiments support clinicians’ preferences for recontouring techniques, few limitations caution against drawing definitive clinical applications:

- Clinically, recontouring is always followed by polishing using strips and disks to eliminate roughness.^2,23^ Had we applied polishing in our experiment, some angulation at the grinded walls of the “teeth” could measure differently.
- Because the “teeth” were shaped to the proximal anatomy of incisors, this sample can only serve to study the changes in proximal contour before and after grinding. For the same reasons, these results cannot be transposed without risk of error for clinical application. Indeed, the approximate proximal shape and the fixation of the teeth with resin in a plaster model can lead to an excess of pressure when grinding, and may affect the results. Clinically, the physiological mobility and the ligament proprioception indirectly control the pressure exerted on the tooth. In future studies, fixing the teeth in a silicone model could solve this problem.^17^
- Finally, shaping the “teeth” in our experiment likely affects its surface texture and precludes a micro-qualitative study of the grinded enamel. Incisors or premolars extracted for orthodontic or peri-odontal reasons should be used in that purpose.^26^

## CLINICAL SIGNIFICANCE

- The use of abrasive strip preserve the best shape of the proximal side.
- Abrasive strip could be the last step of any proximal recontouring technique.

## CONCLUSION

The use of strip alone or in addition to another proximal teeth stripping techniques appears to provide optimal proximal profile although it requires more time. When carried out properly, and in specific circumstances, it meets treatment objectives without compromising the integrity of the dental and periodontal tissues.

This study can be a preamble to more advanced studies to reflect more the clinical reality.

### Why This Paper is Important to Pediatric Dentist?

- Children should be given every opportunity to get the best orthodontic treatment result.
- The pediatric dentist can make a significant contribution during treatment.

### What This Paper adds?

- Considering the importance of the anatomical shape of the incisors, it is important to spread awareness among the dentists regarding the recontouring techniques.
- The principles described in this study can help the clinician in choosing the striping technique in collaboration with the orthodontist for a better result that contribute to the well-being of the child.
